# Structural Response of Steel Jacket-UHPC Retrofitted Reinforced Concrete Columns under Blast Loading

**DOI:** 10.3390/ma14061521

**Published:** 2021-03-20

**Authors:** Mohammad Hanifehzadeh, Hadi Aryan, Bora Gencturk, Dovlet Akyniyazov

**Affiliations:** Sony Astani Department of Civil and Environmental Engineering, University of Southern California, 3620 S. Vermont Avenue, KAP 210, Los Angeles, CA 90089-2531, USA; hanifzadehm@gmail.com (M.H.); haryan@usc.edu (H.A.); dovlet.akyniyazov@hotmail.com (D.A.)

**Keywords:** blast loading, finite element analysis, reinforced concrete, retrofit, UHPC

## Abstract

The lateral capacity of exterior concrete columns subjected to a blast load is the key factor in the building collapse probability. Due to potentially severe consequences of the collapse, efforts have been made to improve the blast resistance of existing structures. One of the successful approaches is the use of ultra-high-performance-concrete (UHPC) jacketing for retrofitting a building’s columns. The columns on the first floor of a building normally have higher slenderness due to the higher first story. Since an explosion is more likely to take place at the ground level, retrofitting the columns of the lower floors is crucial to improve a building’s blast resistance. Casting a UHPC tube around a circular RC column can increase the moment of inertia of the column and improve the flexural strength. In this study, a retrofitting system consisting of a UHPC layer enclosed by a thin steel jacket is proposed to improve the blast resistance of buildings in service. Most of the previous research is focused on design aspects of blast-resistant columns and retrofitting systems are mostly based on fiber reinforced polymers or steel jackets. A validated FE model is used to investigate the effectiveness of this method. The results showed significant improvement both at the component and building system levels against combined gravity and blast loading.

## 1. Introduction

Prevention of progressive collapse due to blast is an important consideration in design of modern buildings and bridges. Columns are the main elements for the stability of a structure and several approaches have been proposed in literature to improve the performance of reinforced concrete (RC) columns under combined axial and extreme lateral loading. One of these approaches is to use ultra high-performance concrete (UHPC) for retrofitting RC columns to improve their blast resistance [1,2]. UHPC is a cement-based composite material with superior mechanical performance compared to ordinary concrete. The compressive and tensile strength of UHPC are 3–5 times as high as those of conventional concrete due to optimized packing, water-reducing admixtures, and steel, polymeric or carbon fibers [3]. UHPC may be used in critical locations of a structure to improve its overall performance under extreme loading scenarios. During an explosion, there is a risk of initial casualties due to direct contact with the blast wave. In addition, there is a secondary and potentially more severe risk due to the collapse of the building [4]. The secondary casualties might be significantly more as it was observed in the Oklahoma City bombing in 1995 where 85% of the deaths were due to the collapse of the building [5]. Effective retrofitting methods could improve blast resistance and reduce secondary casualties in existing buildings.

Several numerical and experimental studies have been conducted to assess composite sections under different loading scenarios. Zhang et al. [6] investigated concrete-filled double-skin (CFDS) tubes and observed superior performance compared to a RC column under near field blast loading. Four circular and seven square columns were tested. Both the square and circular tubes had outer and inner dimensions of 210 mm and 100 mm, respectively, and the thickness of the outside and inside steel tubes was 5 mm. An emulsion explosive equivalent to 1, 17, 35 and 50 kg of trinitrotoluene (TNT) at a standoff distance of 1500 mm was used. It was observed that the specimen without an axial load had 25% larger peak displacement compared to the specimen with 1000 kN of axial load. It was also reported that the hollow area inside the column had an insignificant effect on the overall structural response in terms of the period of oscillation, and the maximum and residual deformations.

Wang et al. [7] evaluated the load resistance and residual strength of eight concrete-filled steel tube (CFST) columns subjected to adjacent blast. Four of the columns were circular with a diameter of 194 mm and the other four had a 200 mm square cross-section. From each group of columns, two had 2.8 mm tube thickness and two had 3.8 mm tube thickness. The two columns with thinner tubes in each group were subjected to a 500 kN axial load while the other two columns were subjected to a 562 kN axial load (40% of the axial load capacity). The standoff distance was 1.5 m for all the tests and the columns were subjected to a blast ranging from 25 to 50 kg equivalent weight of TNT. The results showed a strong dependence of the mid-span deflection on the weight of charge. Increasing the charge weight by 10% tripled the mid-span deflection of the square column. The other important result was regarding the influence of steel tube thickness. Increasing the tube thickness by 1 mm reduced the peak mid-span deflection by 50 and 67% in the square and circular columns, respectively. To assess the residual loading capacity of the columns, two of the circular and two of the square columns were tested under axial loading following the blast load. The axial residual capacity tests revealed that the columns with thicker steel tubes retain a larger residual axial capacity in each group and the square columns have more residual capacity compared to the circular columns.

Li et al. [8] investigated the behavior of CFDS tube columns subjected to a close-range blast. Four large scale experiments were conducted on three columns with 2.5 m height, 159 and 325 mm inside and outside concrete diameter, respectively, and 6 mm thick inside and outside steel tubes. The first and the second tests were conducted on the first column from 300 and 200-mm standoff distances respectively placed at 400 and 500 mm from the footing surface. These tests were intended to investigate the influence of the standoff distance in which a 5 kg weight of TNT charge was used. The columns showed between 10 to 80 mm local indentation with the largest measurement corresponding to the largest TNT charge in the last test. In addition to the significant increase in the indentation, the larger charge caused the steel tube close to the detonation to endure a fracture failure. The steel tube was effective in preventing the concrete from spalling and dissipating the blast energy.

Kyei and Braimah [9] considered the effect of transverse reinforcement detailing according to the Canadian concrete design code [10] and axial load on the blast response of columns. Finite element (FE) models were developed and validated based on the results of the experiments performed by Siba [11]. Three rectangular, 0.3 × 0.3 m configurations, were considered with different stirrup spacing: normal with 300 mm spacing, seismic with 150 mm spacing at both ends, and 75 mm spacing at both ends and the center. It was found that the reduced stirrup spacing does not improve the structural performance in the long-range blast. In addition, under blast loading, the gravity loads from the upper stories of the building resulted in a reduced lateral deformation. However, at high axial load levels, the crushing of concrete and the buckling of longitudinal bars at mid-height was observed.

Omran and Mollaei [12] performed an experimental and numerical investigation on rectangular RC columns made with normal strength concrete. They proposed six different retrofitting schemes based on steel jacketing to improve the blast resistance. The retrofitting schemes included attachment of U-shaped plates, angles and straight plates to the column as discontinuous jacketing along the height in such a way that all the schemes have equal cross section after strengthening. The experiment was performed on 350 × 350 × 3000 mm specimens sustaining an axial load in simply supported conditions. A blast load with a scaled distance of 1.14 m/kg^1/3^ was applied on all four specimens. It was observed that the retrofitting method consisting of two steel channels enclosing the column had the best performance compared to the other configurations.

Wang et al. [13] simulated hybrid fiber-reinforced polymer (FRP) concrete-steel double-skin tubular columns under blast loading using a commercial FE analysis code. In their proposed retrofitting scheme, the outer FRP tube provides confinement to the infilled concrete and the inner steel tube provides flexural strength against blast loading. The authors investigated the effect of several parameters on structural performance. The inner steel tube thickness, hollowness ratio, axial load level, and fiber orientation were found to be more effective than the concrete strength and the outer FRP tube thickness in improving the blast-resistance.

Thai et al. [14], used a commercial FE analysis code to study the behavior of 250 × 250 × 3600 mm rectangular concrete columns retrofitted with a steel jacket under blast loading having scale distances from 0.10 to 0.40 m/kg^1/3^. The effects of the axial force and the steel thickness on the blast performance of the specimens were investigated. It was found that an explosion close to the base of the column causes more severe damage compared to that at mid-height. Increasing the steel thickness from 3 to 6 mm did not prove to be an effective solution to reduce blast damage.

Cui et al. [15] investigated the damage response of two concrete-filled steel tube columns subjected to near-filed blast loading with a scale distance of 0.14 m/kg^1/3^. The column height was 1800 mm, and the charge was 500 mm away from the mid-height of the columns. One of the columns was solid concrete strengthened with a 7 mm thick outer steel pipe having a diameter of 273 mm. The other column was identical to the first one, except for having a hollow section inside, and it was strengthened with a 3 mm inner steel pipe having a 50 mm diameter. The explosive was a 50 kg TNT. The column with a solid section of concrete and outer steel pipe resulted in 40% smaller deformation at mid-height compared to the hollow concrete column with outer and inner steel pipes. The deformations of the columns at the top and bottom were similar for both columns and they were negligible compared to the deformations at mid-height. A summary of the most recent relevant finite element (FE) and experimental (EXP.) research studies are provided in Table 1.

**Figure 1:**
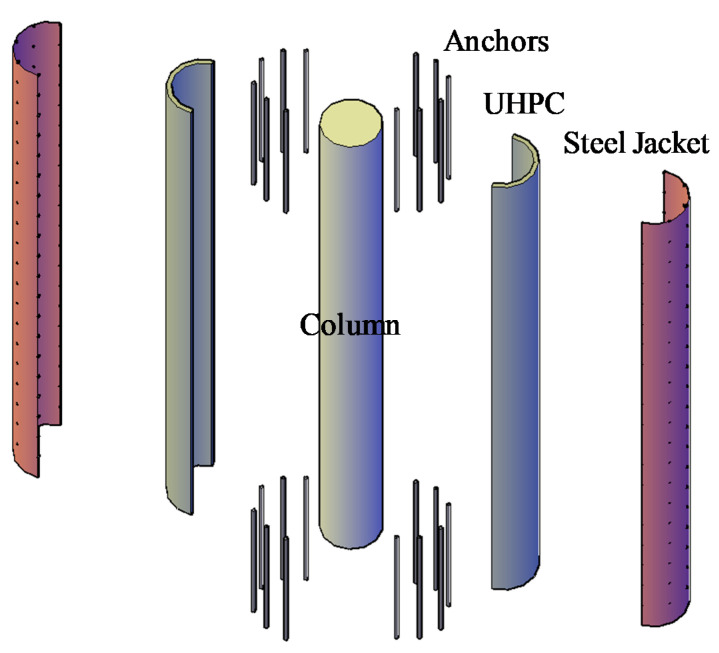
Proposed retrofitting system for existing columns against blast loading. Note the shear studs are attached to the inner surface of the steel jacket.

In this study, as shown in Figure 1, a retrofitting system, consisting of a UHPC layer confined by a steel jacket is proposed to provide increased lateral load resistance against blast loading. This method is introduced to improve the flexural capacity of the RC columns subjected to the blast load. This configuration is called the composite section hereafter. First, holes are drilled in the footing and the slab above and adhesive steel anchors are installed. Then, the steel jacket, made of two half-circular steel tubes, is placed and welded around the existing column and anchors. Then the gap between the jacket and column is filled with UHPC through holes drilled in the tube such that the anchors are embedded in the UHPC.

The perfect composite interaction between the steel and UHPC is obtained by installing studs inside the steel jacket. Several advantages have been reported for this composite section such as high flexural stiffness, improved fire resistance, and enhanced ductility, energy absorption, and stability [16]. Concrete filled tubes are generally efficient because the cost of the rebar cage is eliminated, and faster construction can be achieved. This method is proposed to improve the structural response of columns against axial and lateral loads caused by a blast or an earthquake. The confinement of the UHPC layer could also be provided by FRP composites. However, a steel jacket provides better integrity against projectiles, flying fragmentation, and high temperature in terms of strength and bond loss. In addition, formwork for casting UHPC is not needed in the case of steel jacket and unlike FRP, no hazardous fumes are generated in the presence of flames. As will be shown later in this paper, the proposed method increases the axial and flexural capacity of the column. Additionally, it improves the blast performance by increasing the mass and ductility of the member.

**Figure 2:**
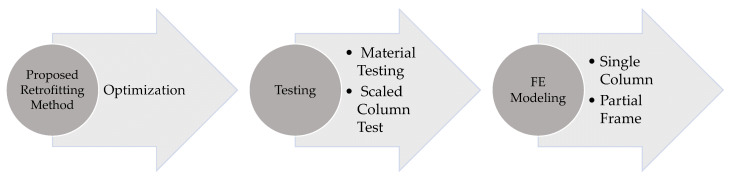
Flowchart of the study.

The structure of this study is presented in Figure 2. 3D nonlinear FE analyses were conducted using ABAQUS/EXPLICIT (Version 6.14) [17] to investigate the flexural and axial capacity of an RC column retrofitted with the proposed approach. Validation of the FE model is performed using a scaled RC column subjected to a cyclic load with a maximum drift ratio of 7.69%. Separate models were prepared for a single column and part of a multi-story building frame. During the analysis, first, an axial service load was applied on the column and then the column was subjected to a near-field blast load. Finally, a vertical displacement was applied on top of the column to obtain the residual capacity of the blast damaged column. The residual capacity is compared to the initial capacity of the column and the loss of the capacity of the non-retrofitted column, caused by the blast, is assessed. Finally, the improvement of the residual capacity due to the presence of the UHPC and steel confinement layers is evaluated.

## 2. Experimental Program

In the experimental program, a scaled column made with normal strength concrete (NSC) was tested under combined axial and lateral loading. Additionally, material tests according to relevant American Society for Testing and Materials (ASTM) International standards were performed on prisms and cylinders made from NSC and UHPC to obtain the mechanical properties of both materials. The data from the material tests were used as inputs for the constitutive models.

### 2.1. Material Properties

**Figure 3:**
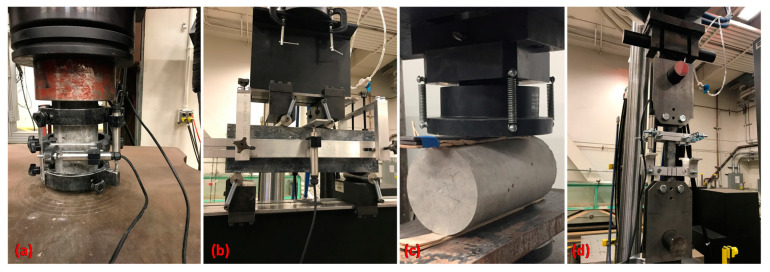
Material tests (**a**) compression, (**b**) flexure, (**c**) split tension, and (**d**) uniaxial tension on NSC and UHPC. Note that uniaxial tension tests were only performed on UHPC samples.

The proportions of the NSC and UHPC mixture developed by the authors are presented in Table 2. To prepare the UHPC, Dramix straight steel microfibers that are 13 mm long and 0.21 mm in diameter (aspect ratio equal to 62) were added to the mixture at a volume ratio of 2.5%. The tensile strength and the modulus elasticity of the steel fibers are 2750 and 200,000 MPa, respectively. The material tests are illustrated in Figure 3 and the results are summarized in Table 3.

### 2.2. Column Testing

**Figure 4:**
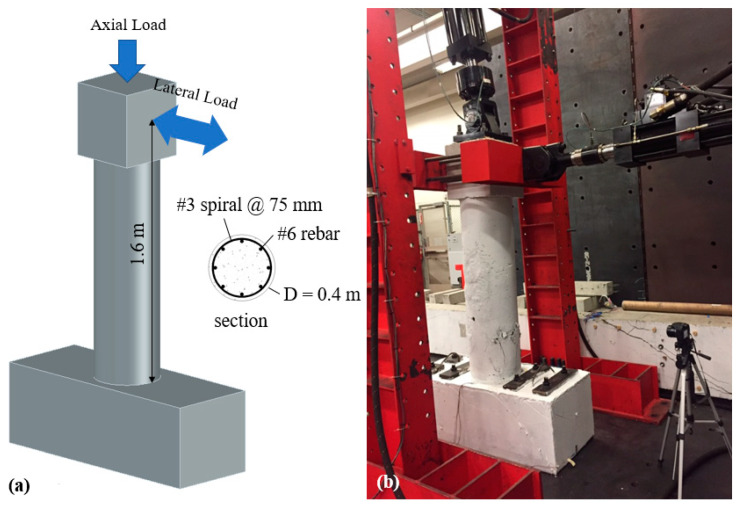
Column test (**a**) geometry and loading configuration and (**b**) test setup.

**Figure 5:**
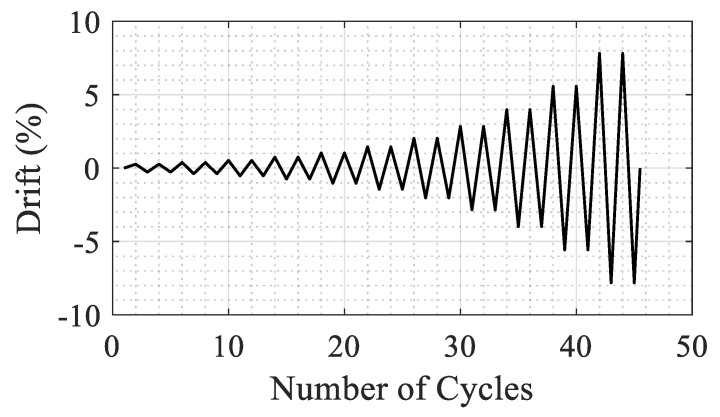
Cyclic loading protocol used in the experiment (maximum drift is 7.69%).

As mentioned earlier, a scaled RC column, shown in Figure 4, was tested under combined axial and cyclic lateral load. The NSC column had an aspect ratio of 4, it was reinforced with U.S. #6 (19 mm diameter) longitudinal rebar (1.75% by volume) and confined with U.S. #3 (9.53 mm diameter) spiral reinforcement at 75 mm spacing (0.46% by volume). The axial load was kept constant at 5% of the axial capacity of the column (or 334 kN) during testing. The lateral load was applied following a quasi-static cyclic loading protocol as shown in Figure 5. The loading protocol included two cycles at each of 0.27, 0.38, 0.52, 0.73, 1.02, 1.42, 2, 2.8, 3.92, 5.48, 7.69% drift ratio levels.

**Figure 6:**
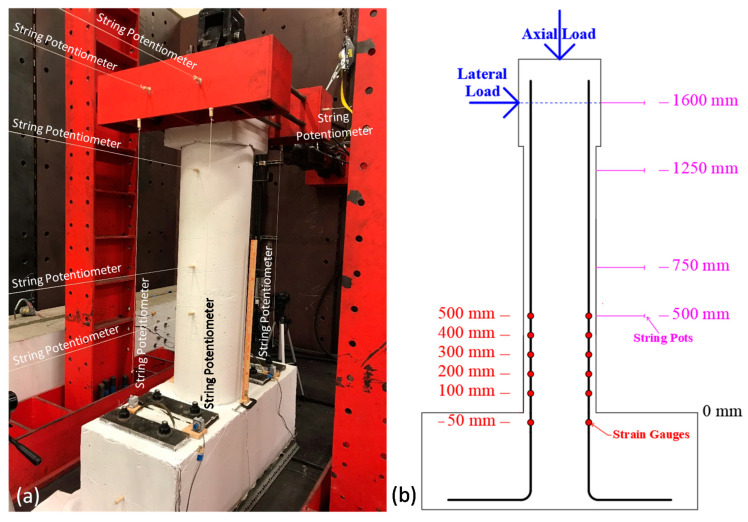
Column instrumentation (**a**) actual and (**b**) schematic with locations.

**Figure 7:**
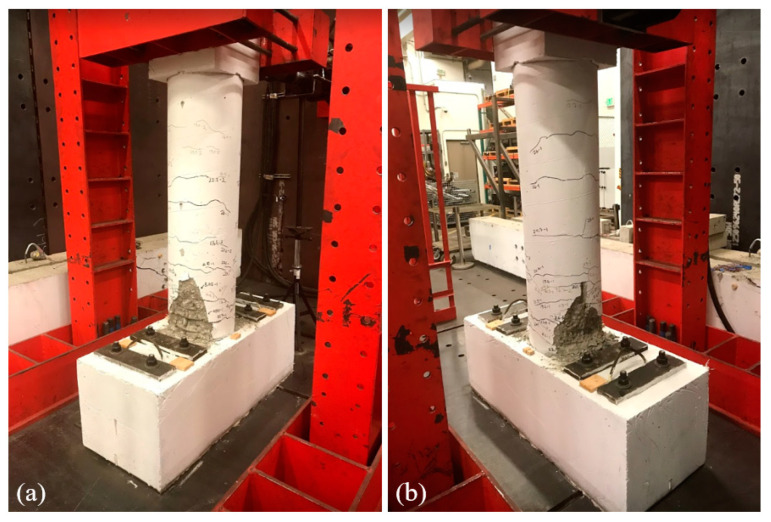
Column condition after testing (**a**) East and (**b**) West view.

**Figure 8:**
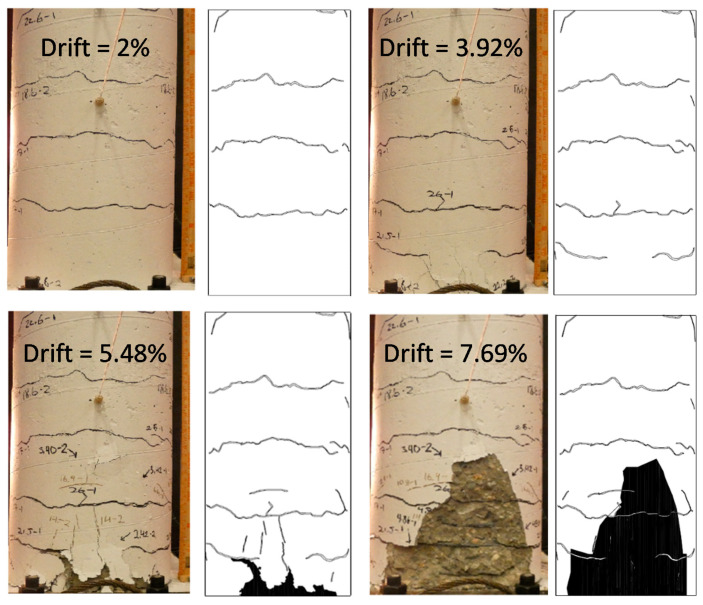
Crack maps at four different column drifts.

As shown in Figure 6a, the deformation of the column was recorded using nine string potentiometers with a 635 mm stroke. In addition to the string potentiometers, 12 strain gauges, shown in Figure 6b, were installed on two of the longitudinal rebar to monitor yielding. The rotation of the column cap was measured to calculate the drift and the horizontal component of the vertical actuator force, and obtain the base shear. The results of the experiment are discussed and compared with the FE model in Section 3.2. The damage and crack patterns after testing are shown in Figure 7 and Figure 8.

## 3. Modeling, Analysis and Discussion

### 3.1. Sectional Analysis

Preliminary parametric analyses were performed using CSICOL [22] software to evaluate the effect of the steel and UHPC thicknesses on the overall performance of the column. Based on the results, values for the thicknesses are determined before more computationally intensive 3D FE simulations are conducted in Section 3.2.

**Figure 9:**
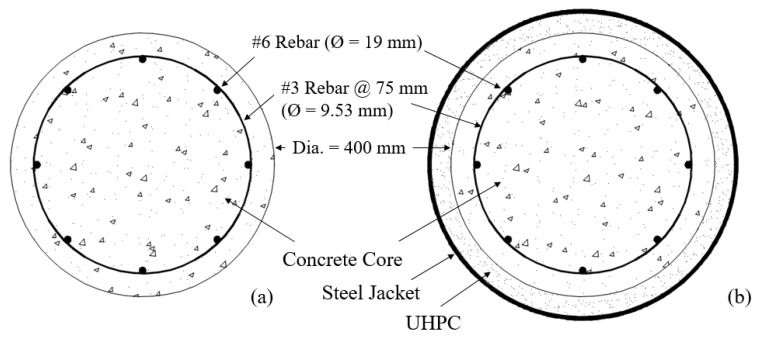
Model configuration (**a**) RC and (**b**) UHPC retrofitting with a steel jacket.

The calculation of axial and flexural capacity is based on ACI 314 [23]. A rectangular distribution of stresses was used for the NSC. The software assumes no slip between the steel and UHPC, or steel and NSC. Therefore, the results from the software are an upper bound to the flexural capacity. In fact, the studs welded to the steel jacket prevent slippage between the steel and UHPC; however, some relative displacement may be observed between the UHPC and NSC core at high drift levels. Two separate models with the cross-sections called RC and composite sections (shown in Figure 9) were developed for analysis. The difference between the two models is the presence of the UHPC layer and the steel jacket in the composite section.

**Figure 10:**
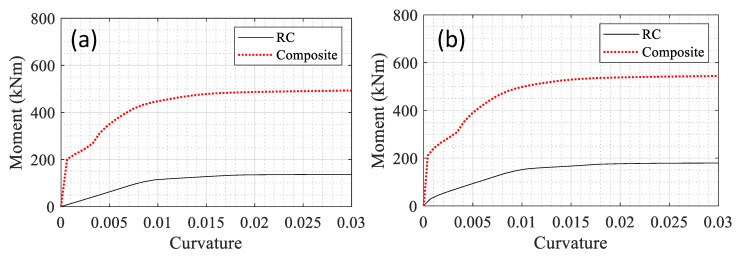
Comparison of the moment-curvature diagrams for the two sections under (**a**) no axial load and (**b**) under 5% axial load.

The moment-curvature diagrams for the RC and composite sections are compared in Figure 10a,b without and with the axial load, respectively. In the experiment, a 334 kN axial load (about 5% of the ultimate capacity) was applied to the specimen. The same load was applied to the model in the software for consistency. The maximum moment in the RC and composite section were obtained as 138 and 495 kNm, respectively, under no axial load. These values were calculated as 179 and 543 kNm under axial loading.

**Figure 11:**
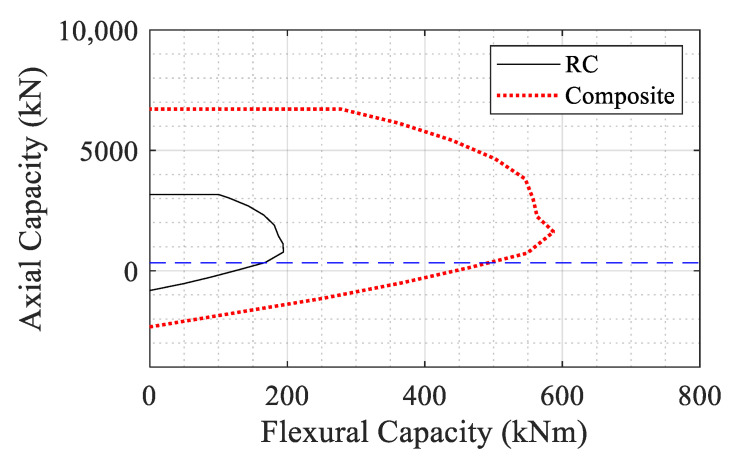
Interaction diagrams for RC and composite column sections.

The interaction diagram for the RC and the composite sections are compared in Figure 11 where a significant improvement in the flexural capacity is observed. The horizontal line in Figure 11 corresponds to the 5% axial service load of the RC section.

**Figure 12:**
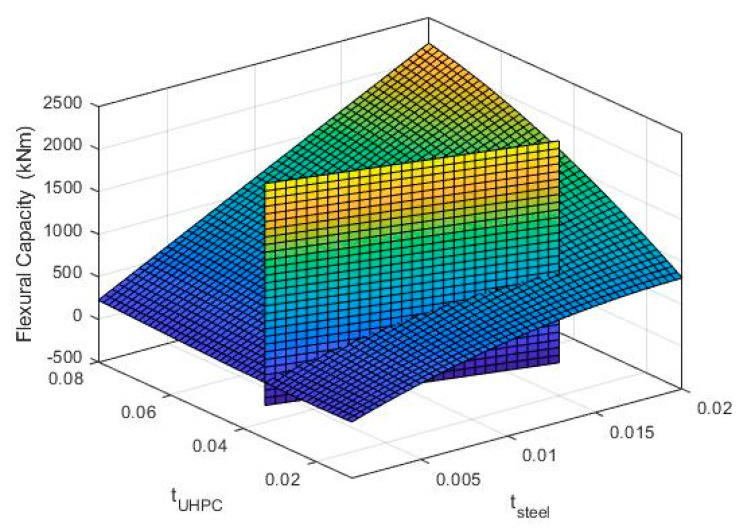
Flexural capacity as a function of steel and UHPC thicknesses.

Several analyses with different steel and UHPC thicknesses were performed to understand the effect of the retrofit geometry on the overall flexural capacity. The thickness of the steel tube, *t*_steel_, and the thickness of the UHPC layer, *t*_UHPC_, were varied in the ranges: 20 mm < *t*_UHPC_ < 80 mm and 5 mm < *t*_steel_ < 20 mm. A surface was determined using the Surface Fitting function in MATLAB R2019b [24] considering zero axial load. Figure 12 shows the flexural strength of the retrofitting system as a function of steel and UHPC thicknesses. It is preferable not to increase the retrofitted diameter of a column drastically not to disrupt the use of the building. Therefore, the maximum combined thickness of 35 mm is considered for the retrofitting layer (shown as the vertical surface in Figure 12). According to Figure 12, compared to the UHPC layer, the steel jacket is more effective in improving the flexural capacity. However, the total cost of retrofitting is highly affected by the steel thickness considering the cost for rolling, welding and ease of handling. Therefore, a thickness of 5 mm is used for the steel and consequently, 30 mm is selected for UHPC.

### 3.2. Finite Element Model Validation

In this section, a more advanced non-linear FE analysis using representative material models is conducted with the dimensions determined for the retrofit in the previous section. The concrete damage plasticity (CDP) model is used for modeling NSC and UHPC [25]. The model considers the degradation of the elastic stiffness caused by plastic straining both in tension and compression. More details about the model are available in the ABAQUS user manual and literature [17,26,27]. The material properties and input parameters for the CDP model for the NSC and UHPC are presented in Table 4. The stress-strain data points obtained from the experiment were fitted with the constitutive equation for CDP model in Abaqus. The elastic modulus obtained from this fit was slightly different than that obtained in the experiment as specified in the standards.

An elastic-perfectly plastic model with isotropic strain hardening was used for the steel reinforcement and the steel jacket. The input parameters for the model are summarized in Table 5.

**Figure 13:**
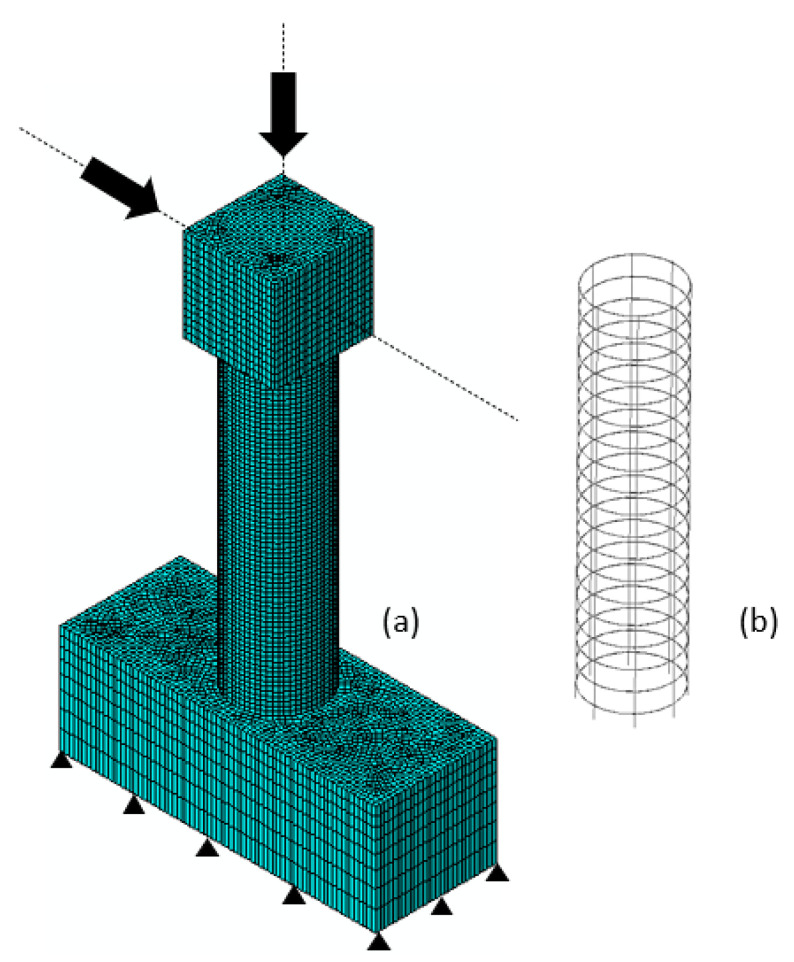
Finite element mesh of models (**a**) NSC and (**b**) reinforcement.

The FE model, shown in Figure 13, was developed with the same geometry as in the experiment. All degrees of freedom were fixed at the bottom of the model. A perfect bond was created between the NSC and the steel reinforcement using the “embedded” command in ABAQUS [17]. Solid C3D8R elements, which are eight node brick elements with quadratic shape functions and reduced integration, were used for the NSC. The reinforcement was modeled using two-dimensional truss elements (T3D2). The total number of elements in the column (except the top and bottom caps) was 28,400 including 1580 rebar elements. Considering the non-linear behavior and the expectation of large deformations in a short period, an explicit integration scheme was adopted.

**Figure 14:**
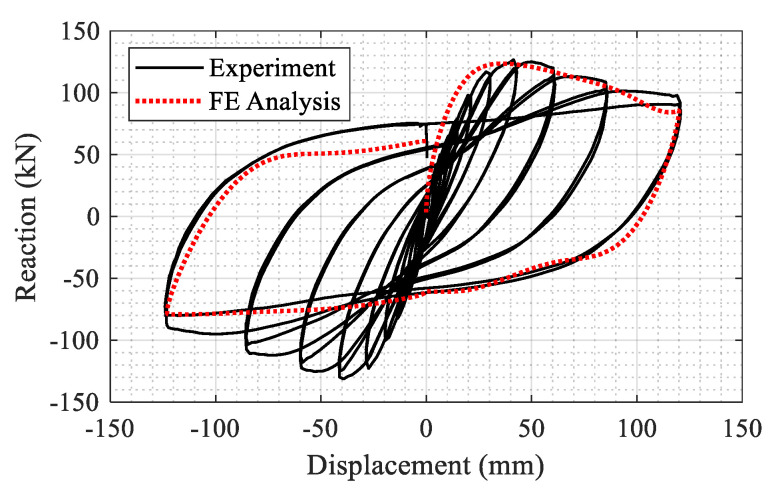
Comparison of experiment and finite element model results.

As a result of the high computational cost of explicit analysis, only the last full cycle with 120 mm peak displacement in each direction was simulated in the FE model. The results from the column testing are compared with the FE model in Figure 14. As seen, the model accurately captures the lateral capacity, and strength and stiffness degradation observed in the experiment.

**Figure 15:**
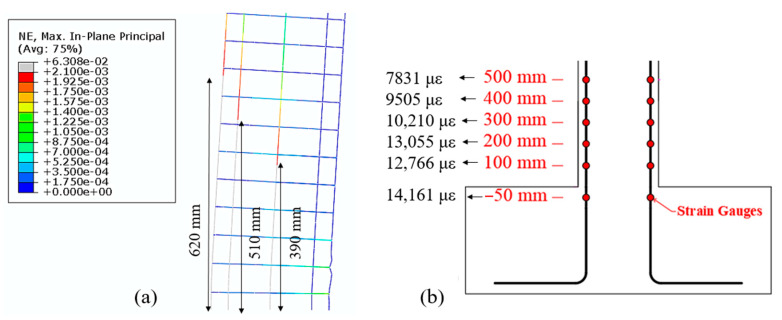
(**a**) Nominal strain from the FE model. Gray color in the legend indicates a strain larger than 2100 µε, which is assumed to be the yield strain of steel. (**b**) Recorded strain obtained from strain gages in the vertical rebar.

Next, the depth of the plastic hinge is estimated from the FE model and it is compared with the results from the experiment. The portion of the rebar in the FE model that has yielded is shown in Figure 15a. The maximum strain readings at the locations of the strain gages are shown in Figure 15b. All the strain gauges in the experiment installed as high as 500 mm above the foundation showed yielding. The estimated depth of plastic hinge from the FE model is 620 mm, which agrees well with the observed data from the experiment.

**Figure 16:**
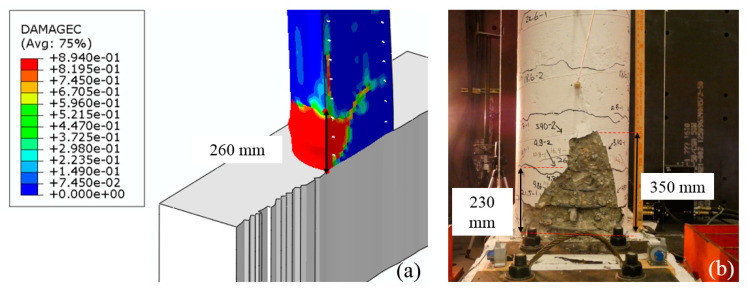
Comparison of (**a**) compressive damage from the FE model with (**b**) spalling in the experiment.

Finally, the depth of spalling in the finite element model is compared with the experiment in Figure 16. A reasonable agreement is observed between the experiment and the computer simulation.

### 3.3. Blast Loading Simulations

**Figure 17:**
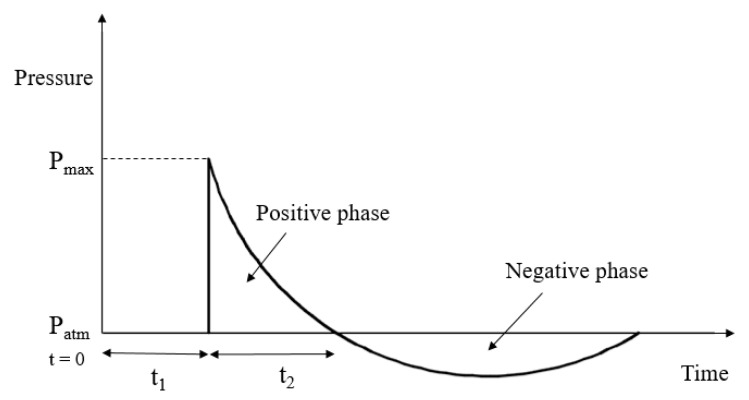
Variation of pressure with time: *t*_0_ is the time of detonation and t_1_ is the time of arrival.

An explosion involves chemical reactions, which cause a rapid increase in the temperature and pressure of the atmosphere surrounding the explosion source of the detonated products. The pressure wave travels away from the source with a spherical front in the radial direction at high velocity (see Figure 17). The blast load is a function of the distance from the source, *R*, and the equivalent charge weight, *W*, in terms of TNT weight. Conversion factors are available to obtain the equivalent TNT weight of other explosive materials. The intensity of a blast load is commonly normalized to a scaled distance, *Z*, which is the ratio of standoff distance to the cube root of the charge weight. A blast load with a scaled distance above 5.88 m/kg^1/3^ corresponding to 5 kg TNT at 10 m distance causes significant deformation and immediate failure of the column considered in this study. Therefore, a scaling distance higher than 5.88 m/kg^1/3^ was found inapplicable for the FE analysis. The pressure as a function of time is obtained using Friedlander’s equation [30] as

(1) $$P\left(t\right)=P_{max}\left[1-\frac{t}{\Delta t}\right]\exp\left[A\times \frac{t}{\Delta t}\right]$$

where *P*(*t*) is the overpressure in kPa as a function of time, *t*; *P_max_* is the maximum pressure in kPa; Δ*t* = *t*_2_ − *t*_1_ is the positive phase duration in ms, and *A* is a dimensionless negative wave decay parameter.

The variation of temperature due to the explosion is not considered in this study and the pressure-time history is applied to the column using the Conventional Weapons Model (CONWEP) built-in ABAQUS [17]. The model introduced by Kingery and Bulmash [31] calculates the pressure at each time step for all nodes in the predefined target surface using Equation (1). The charge amount is defined in equivalent TNT and the explosion source is selected by the user before the analysis. The program calculates the decay coefficient, correct distance, and angles of incidence based on the input parameters.

In this section, the behavior of a single column under blast loading is investigated. To represent a more realistic condition, the height of the column was increased to 4 m while the diameter was kept at 0.4 m similar to what is presented above. Due to a limitation in our experimental setup, the length of the column was restricted to 1.8 m. In the finite element analysis, the length of the column was increased to 4 m. Since the material properties (including steel, rebar and concrete) and all geometries except the length in the FE model remained the same as those in the experiment, the scaling procedure was not applicable and thus was not considered. All the degrees of freedom were fixed for both the foundation and the end cap of the column except the vertical displacement at the top. In the first phase of the analysis, the column was subjected to a monotonically increasing axial loading until failure. This loading scenario is important for the columns away from the blast incident where the axial load increases due to a potential loss of a column due to the explosion. The effect of the volumetric ratio of the confining steel on the axial capacity and ductility of concrete columns has been extensively studied in literature [32,33]. Confinement increases absorbed energy by the concrete core by providing additional strain energy of the yielding hoop steel reinforcement.

**Figure 18:**
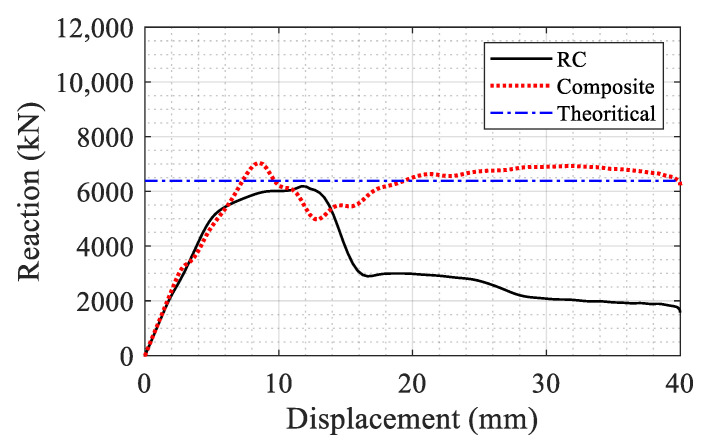
Comparison of the axial capacity of the RC and composite sections.

The axial capacity of the two columns (RC and composite with the proposed retrofit approach) is compared in Figure 18. The composite section showed about 14% increase in the peak axial capacity in compression (7045 versus 6180 kN). The results also showed a larger post-peak residual capacity up to 40 mm axial displacement. The residual axial capacity of the composite section was higher than that of the RC section. The residual strength of the composite column was 3.7 times that of the RC column at 40 mm displacement. The increase in the strength is due to the added axial bearing capacity of the retrofitting layer and the NSC core strength gain due to confinement provided by the retrofitting system. The horizontal line in Figure 18 is the nominal axial capacity of the RC column equal to 6400 kN calculated based on ACI 318 [34].

**Figure 19:**
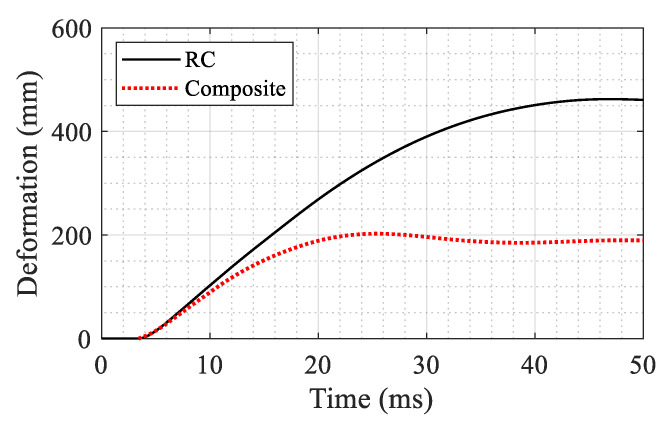
Comparison of the mid-height lateral deformation of the columns under blast loading (with no axial load).

In the second phase of the analysis, the column was subjected to a blast load with a charge of 5 kg of TNT and the source was located at 10 m standoff distance and 1 m above the base of the column. In this phase, the goal is to evaluate the flexural behavior; therefore, no axial load was applied. The boundary conditions were kept identical to the axial loading simulation described above. The lateral displacement at mid-height of the column is taken as an indicator of the blast resistance. The results of the analysis are compared in Figure 19. It is seen that the maximum deformation in the RC column is 2.29 times that of the composite column (202 versus 462 mm).

**Figure 20:**
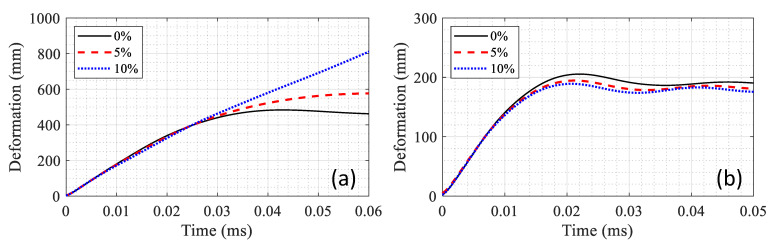
Effect of axial load (percent of maximum axial capacity) on lateral deformation of (**a**) RC and (**b**) composite columns.

In addition, the effect of axial load is compared between the RC and composite sections in Figure 20. Three cases of 0, 5 and 10% axial load of the maximum capacity were considered, and the columns were subjected to the blast load. In the case of RC section, increasing the axial load increased the lateral deformation. 10% axial load resulted in the total failure of the RC column while the presence of the axial load reduced the lateral deformation in the composite section (compare Figure 20a with Figure 20b). The composite column with a 10% axial load experience 189 mm deformation in the middle whereas the deformation was 205 mm for the same with zero axial load. Therefore, it could be concluded that the axial load can improve the structural performance of the composite section under blast loading as long as it is not excessive to cause large second order effects.

**Figure 21:**
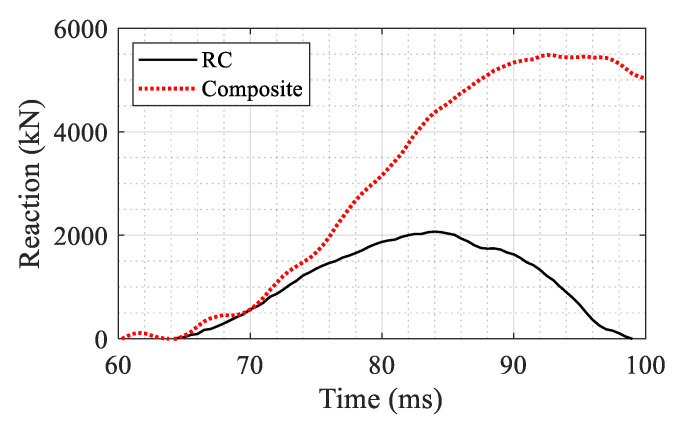
Comparison of the residual axial capacity of the blast damaged columns.

In the final step of the analysis, an axial displacement was applied on top of the blast damaged columns to obtain the residual strength. The residual capacity is defined as the maximum axial load that could be sustained by the column after experiencing the blast load. The maximum residual capacities of the RC and composite sections were obtained as 2034 and 5485 kN, respectively, as shown in Figure 21. The drastic improvement in the case of the composite column is explained by the fact that the UHPC layer protects the core by minimizing the lateral deformation and associated damage during the blast. It also provides additional capacity by providing confinement for the core in the subsequent axial loading.

### 3.4. Progressive Collapse Simulations

**Figure 22:**
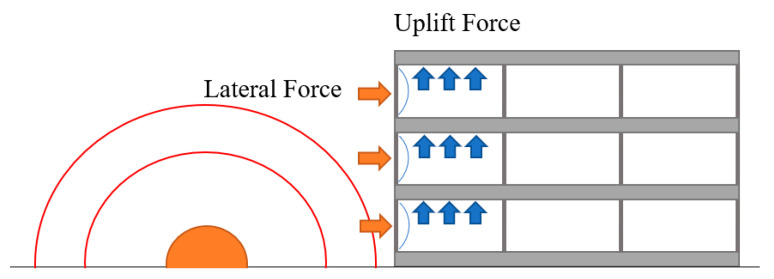
Lateral and uplift pressure caused by a blast incident.

**Figure 23:**
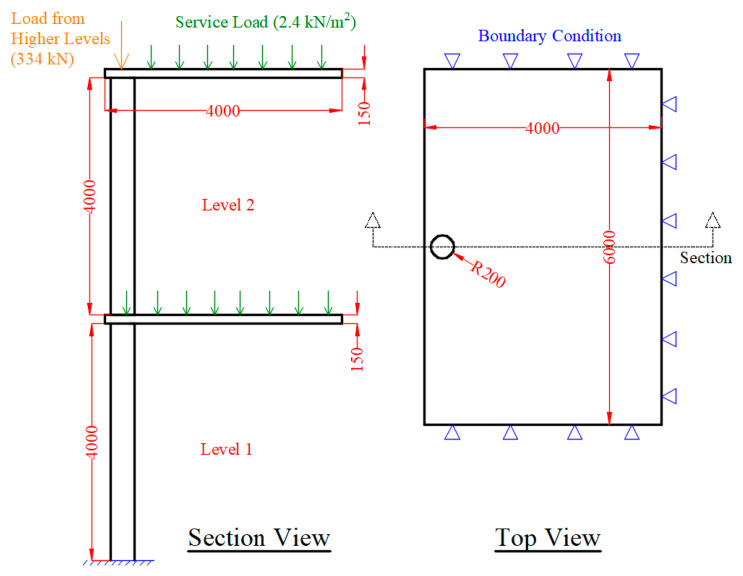
Geometric details and loading and boundary conditions of the model (all dimensions in mm).

**Figure 24:**
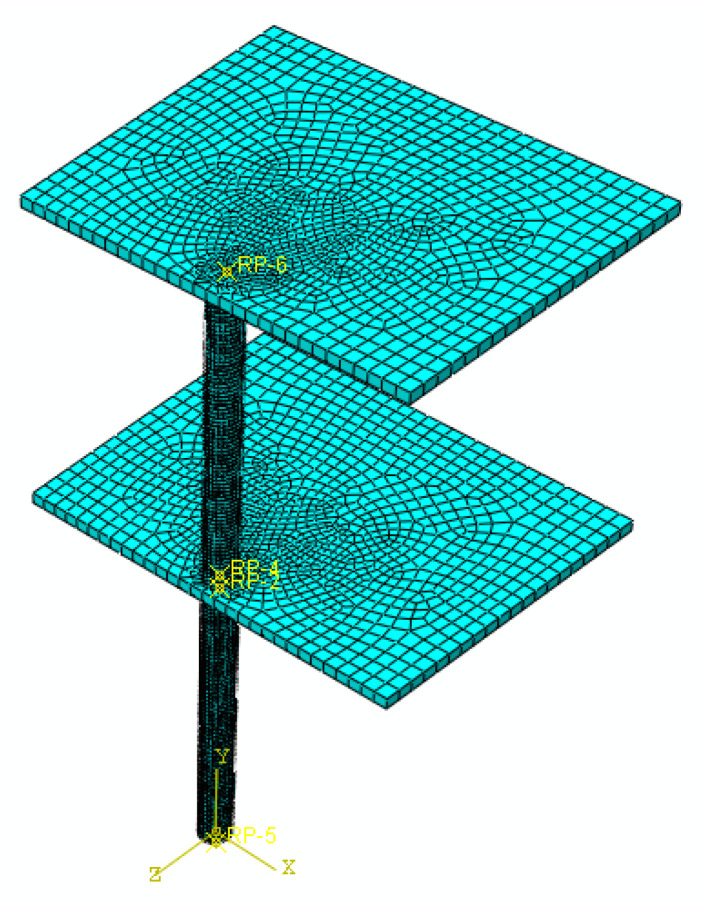
Mesh density of the finite element model.

In this section, a part of a concrete frame consisting of two columns and two slabs was developed for detailed analysis. Although the previous analyses on a single column provide insights about the structural behavior, this configuration is more realistic since a blast induces uplift forces due to the pressure in the vertical direction applied to the bottom of a slab in a building (see Figure 22). In addition, the presence of the slab provides a more realistic boundary condition for the column. In the previous section, the axial load was only applied to the NSC core and the retrofit layer provide confinement and flexural strength. In the frame model, the contribution of the retrofit layer in the axial capacity of the system is also added. For this purpose, a flat slab with 150 mm thickness was considered in the structural system. Linear elastic properties were assigned to the slab concrete. The live load for an office space as per ASCE 7 [35] for the tributary area of the column was combined with the dead load of the slab and applied as downward pressure on the column. The load from the higher floors was also added as a concentrated axial load to the top of the second story column. The geometry and the loading and boundary conditions of the system are shown in Figure 23. The blast source was placed 10 m away from the column in the first story without any offset. The column was fixed at the bottom and the lateral displacement of the slabs in the two in-plane directions was restrained. It is assumed that the specimen is a part of a large building; therefore, considering the small blast charge, the horizontal displacement of the slab is ignored. The FE mesh of the system consists of 59,300 elements as shown in Figure 24. A second model was also developed with a similar geometry except that the retrofitting system with a 30 mm UHPC layer and a 5 mm steel jacket was added.

**Figure 25:**
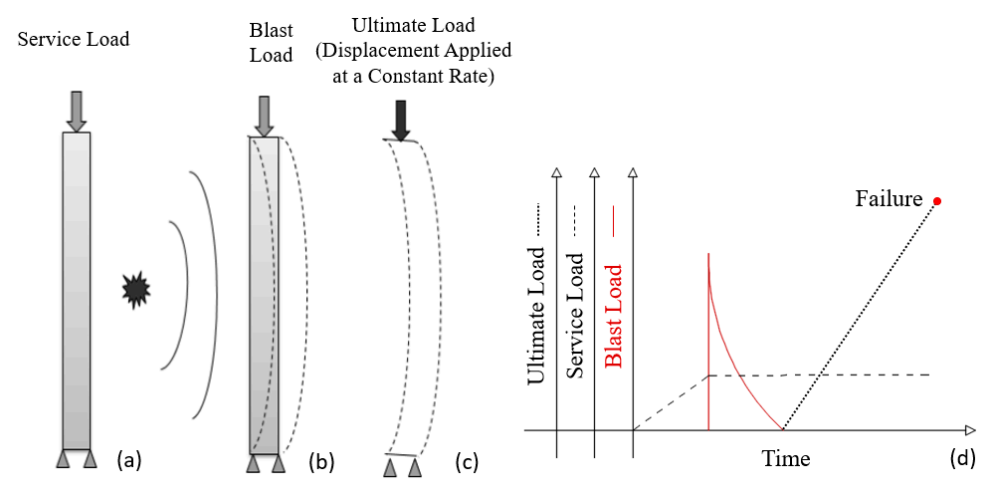
Application of (**a**) service load, (**b**) blast load, (**c**) ultimate load, and (**d**) illustration of the load protocols applied in the FE analysis.

The loading protocol during the blast analysis is shown in Figure 25. The system was loaded in three steps. In the first step, a service load equal to 2.4 kN/m^2^ (office area) pressure in normal direction was applied to both slabs. The axial load from higher floors was considered 5% of maximum axial capacity. Summing up the load from the higher floors, the slab weight and the service load, the total applied load on the first story column is 5.3% of the column’s axial load capacity. In the second step, the blast load was applied. In the final step, the axial load in the column was linearly increased up to the failure of the system. Thereby, the residual capacity of the system after the blast load was estimated for the RC and the composite column systems.

**Figure 26:**
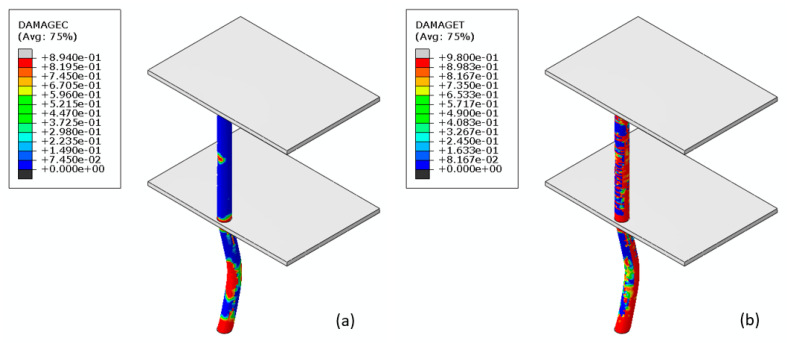
(**a**) Compressive and (**b**) tensile damage in the columns for the RC system.

**Figure 27:**
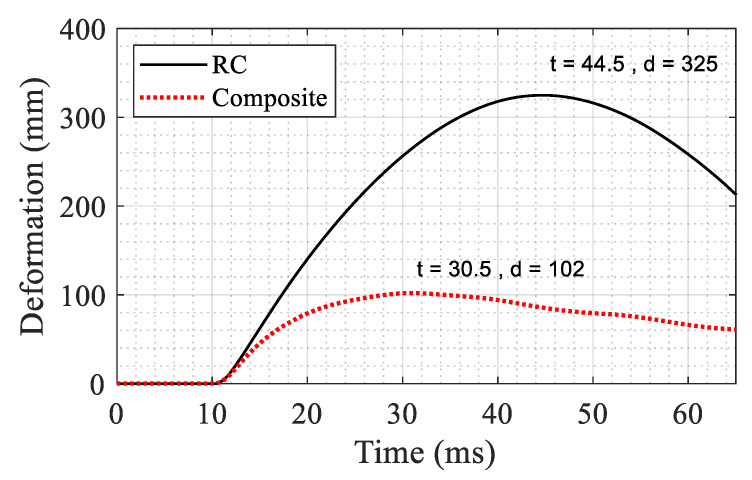
Comparison of deformation time history at mid-height of the first floor (*t* is time in ms and *d* is deformation in mm).

**Figure 28:**
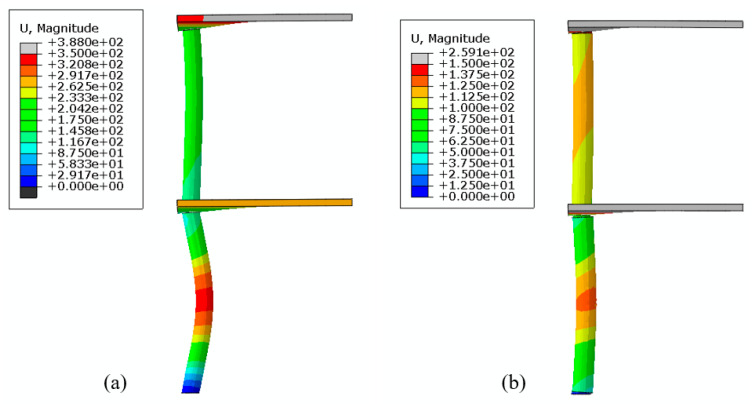
Comparison of the horizontal displacement fields at the maximum displacement (**a**) RC (*t* = 29 ms) and (**b**) composite section (*t* = 46 ms). The legends show the horizontal displacement in mm.

The compressive and tensile damage in the RC system at maximum deformation is shown in Figure 26. The deformation at mid-height of the first floor column is compared respectively in Figure 27 and Figure 28 for the RC and composite systems. It is seen that the maximum horizontal deformation at the mid-height of the column in the first story in the RC system is 3.19 times that in the composite system.

**Figure 29:**
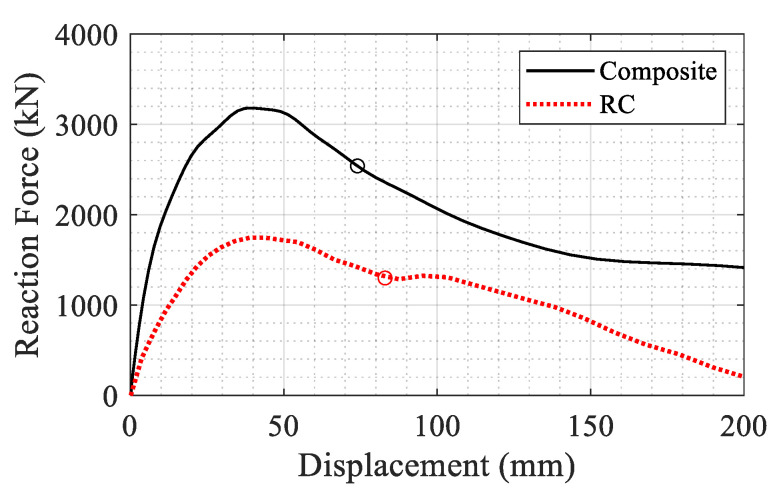
Comparison of residual axial capacity after blast damage between the two systems.

The residual axial capacity of the systems after the blast is shown in Figure 29. The force is the reaction at the lower column and the displacement is the vertical displacement at the top of the upper column. The maximum axial capacity was obtained as 3180 kN for the composite section and 1620 kN for the RC section. It could be concluded from Figure 29 that the retrofit system could increase the capacity of the damaged column by a factor of 1.96 (from 1620 to 3180 kN).

**Figure 30:**
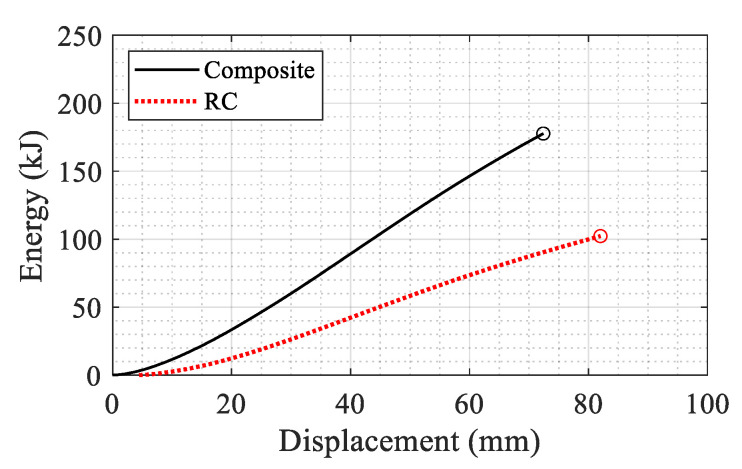
Cumulative dissipated energy for the RC and composite systems after blast damage.

The same pattern is observed as for the results in Figure 21 for the single column. Furthermore, the dissipated energy of each system is calculated as the area under each curve up to a displacement corresponding to a 20% reduction in the maximum reaction force in the post-peak regime. The corresponding displacements are determined as 74 and 83 mm for the composite and RC columns respectively (shown with circles in Figure 29). The dissipated energy for both columns is shown in Figure 30. The composite section showed 1.74 times more energy dissipation capacity compared to the RC section up to the 20% reduction limit.

**Figure 31:**
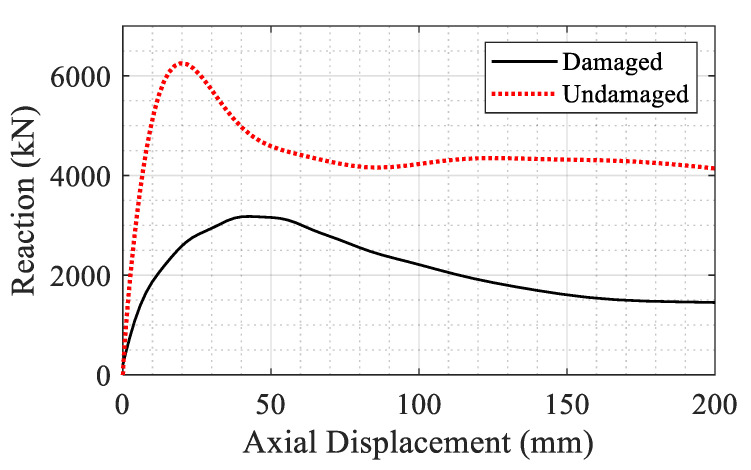
Comparison of axial capacity of the composite system with and without blast damage.

In the final part of the study, the effect of blast on the axial capacity of the frame was evaluated. The retrofitted frame was subjected to an increasing axial load with and without the blast damage. Results (see Figure 31) showed that the composite section can tolerate up to 6250 kN before the damage and after the damage, the capacity is reduced to 3180 kN. This reduction could be attributed to tension developed in the column from the uplift of the floors and also the flexural deformation resulting from the lateral drift.

## 4. Conclusions

In this study, non-linear explicit analysis has been performed to investigate the structural performance of reinforced concrete columns retrofitted with a UHPC layer and a steel jacket under lateral loading. Material tests were performed for calibration of the constitutive models. The FE model was validated using experimental data from a scaled column. The effect of the blast load on the lateral deformation and residual axial capacity was investigated. The following key conclusions are drawn from the results obtained.

- The confinement provided by the UHPC and steel layer increased the peak and residual capacity of the undamaged column. The residual capacity of the composite section was higher by a factor of 3.7 in the uniaxial compression loading scenario at 40 mm axial deformation.
- The investigated retrofitting method was found to improve the residual axial capacity of the column subjected to the blast load by protecting the NSC core from the plastic hinging at mid-height. In the case study, for a 400 mm diameter single column, a 30 mm UHPC layer and a 5 mm steel jacket, the axial capacity of the column was increased by a factor of 2.70. The maximum deformation in the mid-height of the RC column was 2.29 times that of the composite column.
- Considering the results from the detailed frame model, the residual strength of the composite column with the service load equal to 5.3% of the ultimate axial capacity was 1.96 times that of the RC column and the energy dissipation capacity was 1.74 times that of the RC column. The lateral displacement in the RC column at mid-height of the first story was 3.19 times that in the composite section.
- The results also showed that the application of axial load can reduce the lateral displacement of the column under blast load and thus the marginal extra weight from the retrofitting system is beneficial to the structural performance.

## Figures and Tables

**Table 1:** Summary of recent studies on strengthening of columns against blast loading.

No.	Study	Year	Methodology	Retrofit Scheme
1	Cui et al. [15]	2020	FE ^1^	Steel jacket
2	Thai et al. [14]	2020	FE	Steel jacket
3	Li et al. [8]	2019	FE + EXP. ^2^	Concrete-filled double-skin tube
4	Wang et al. [13]	2018	FE	FRP-concrete-steel tube
5	Wang et al. [7]	2017	EXP.	Steel jacket
6	Omran and Mollaei [12]	2017	FE and EXP.	Steel jacket
7	Kyie and Braimah [9]	2017	FE	RC with improved stirrup spacing
8	Zhang et al. [6]	2016	EXP.	Concrete-filled double-skin tube

^1^ FE: finite element analysis. ^2^ EXP.: experimental.

**Table 2:** Mixture proportioning of UHPC.

Components	Weight Ratio		Max. Particle Size
	UHPC ^1^	NSC ^2^	(µm)
Cement (Type I/II)	1.00	1.00	200
SF Densified (Gry from Norchem)	0.25	0.00	20
Silica Powder (SCS40 from US Silica)	0.25	0.00	250
Fine Sand (L60 from US Silica)	0.40	1.20	600
Coarse Sand (GS#22 from US Silica)	0.60	2.00	2000
Water	0.27	0.40	-
HRWR ^3^ (Sikament 2110)	0.02	0.00	-

^1^ UHPC: ultra high-performance concrete. ^2^ NSC: normal strength concrete. ^3^ HRWR: high range water reducer.

**Table 3:** Material properties of NSC and UHPC.

Property	NSC ^1^	UHPC ^2^	Standard
Compressive Strength (MPa)	42	146.2	ASTM C39 [18]
Split Tensile Strength (MPa)	4.3	15.1	ASTM C496 [19]
Direct Tensile Strength (MPa)	N.A. ^3^	10.2	N.A.
Modulus of Rupture (MPa)	4.1	17.6	ASTM C78 [20]
Modulus of Elasticity (GPa)	27	41	ASTM C469 [21]

^1^ NSC: normal strength concrete. ^2^ UHPC: ultra high-performance concrete. ^3^ N.A.: not available.

**Table 4:** Parameters of the CDP model [15,28].

Parameter	Notation	NSC ^1^	UHPC ^2^
Dilation Angle	Ψ	38	10
Flow Potential Eccentricity	ϵ	0.1	0.1
Biaxial/Uniaxial Compression Plastic Stress Ratio	*f*_b0_/*f*_c_	1.16	1.10
Second Stress Invariant Ratio	Κ_c_	0.667	0.667
Viscosity Parameter	µ	0.0001	0.0001
Density (kg/m^3^)	ρ	2400	2500
Poisson’s Ratio	γ	0.19	0.2
Modulus of Elasticity (GPA)	*E*	31	39
Compressive Strength (MPa)	*f’_c_*	42	146

^1^ NSC: normal strength concrete. ^2^ UHPC: ultra high-performance concrete.

**Table 5:** Material properties of steel jacket and rebar.

Section	Yield Stress (MPa)	Modulus of Elasticity (MPa)	Yield Strain	Ultimate Stress (MPa)	Ultimate Strain
	*f_y_*	*E_s_*	*ε_y_*	*f_u_*	*ε_u_*
Steel Jacket (ASTM A36 [28])	250	200,000	0.0013	400	0.20
Reinforcement (ASTM A615 [29])	420	200,000	0.0024	620	0.14

## Data Availability

The data presented in this study are available upon reasonable request from the corresponding author.
